# Quaternion-Based Attitude Estimation of an Aircraft Model Using Computer Vision

**DOI:** 10.3390/s24123795

**Published:** 2024-06-12

**Authors:** Pavithra Kasula, James F. Whidborne, Zeeshan A. Rana

**Affiliations:** Centre for Aeronautics, Cranfield University, Cranfield MK43 0AL, UK; pavithra.kasula@cranfield.ac.uk (P.K.); zeeshan.rana@cranfield.ac.uk (Z.A.R.)

**Keywords:** computer vision, extended Kalman filter, inertial measurement unit, quaternions, Euler angles, computer-aided design, dynamic wind tunnel testing, flight dynamics

## Abstract

Investigating aircraft flight dynamics often requires dynamic wind tunnel testing. This paper proposes a non-contact, off-board instrumentation method using vision-based techniques. The method utilises a sequential process of Harris corner detection, Kanade–Lucas–Tomasi tracking, and quaternions to identify the Euler angles from a pair of cameras, one with a side view and the other with a top view. The method validation involves simulating a 3D CAD model for rotational motion with a single degree-of-freedom. The numerical analysis quantifies the results, while the proposed approach is analysed analytically. This approach results in a 45.41% enhancement in accuracy over an earlier direction cosine matrix method. Specifically, the quaternion-based method achieves root mean square errors of 0.0101 rad/s, 0.0361 rad/s, and 0.0036 rad/s for the dynamic measurements of roll rate, pitch rate, and yaw rate, respectively. Notably, the method exhibits a 98.08% accuracy for the pitch rate. These results highlight the performance of quaternion-based attitude estimation in dynamic wind tunnel testing. Furthermore, an extended Kalman filter is applied to integrate the generated on-board instrumentation data (inertial measurement unit, potentiometer gimbal) and the results of the proposed vision-based method. The extended Kalman filter state estimation achieves root mean square errors of 0.0090 rad/s, 0.0262 rad/s, and 0.0034 rad/s for the dynamic measurements of roll rate, pitch rate, and yaw rate, respectively. This method exhibits an improved accuracy of 98.61% for the estimation of pitch rate, indicating its higher efficiency over the standalone implementation of the direction cosine method for dynamic wind tunnel testing.

## 1. Introduction

Flight dynamics models are essential for designing new aircraft, as they are used for assessing handling qualities, designing flight control systems, and conducting flight simulations. These tasks are crucially performed during the early stages of aircraft design. Additionally, the construction of sub-scale flight demonstrators offers an invaluable means to evaluate designs early in the design cycle, as well as to validate models and facilitate model development [1]. Furthermore, they can be used for model parameter identification. Approaches to flight dynamics identification encompass theoretical and numerical analyses, wind tunnel testing, and flight testing. In the initial design phase, theoretical parameter estimation is common but may have limited accuracy, necessitating wind tunnel testing. This method is comparatively lower in cost and requires less time to manufacture than a proof-of-concept sub-scale flying vehicle. Sub-scale models can also be tested in wind tunnels to extract aerodynamic and flight dynamics data.

While static wind tunnel testing suffices for identifying the main parameters such as lift and drag, non-linear flight regimes involving high angles of attack demand investigation into non-steady aerodynamics. Dynamic wind tunnel (DWT) testing serves this purpose [2]. Although dynamic wind tunnel testing is not yet widespread, advances in model construction methods such as 3D printing and instrumentation are making dynamic wind tunnel experiments more accessible and affordable, thus enriching the design process.

Several DWT testing techniques have been developed to determine the forces acting on the model, including captive tests (such as forced oscillation and rotary balance tests), single-degree-of-freedom (DOF) tests (like free-to-pitch or free-to-yaw, and free-to-roll tests), and free-flying tests. Captive tests aim to measure damping and rotary derivatives but involve a support mechanism that restricts free motion, differing from real flight conditions. One-DOF tests, on the other hand, are utilised to measure dynamic stability derivatives and free-motion modes, as well as evaluate unsteady aerodynamic effects on the model motion. These tests involve the model being supported with an initial condition allowing for free motion. Free-flying tests are further categorised into low-speed tests with a power system and high-speed tests without a power system [3,4].

**Figure 1:**
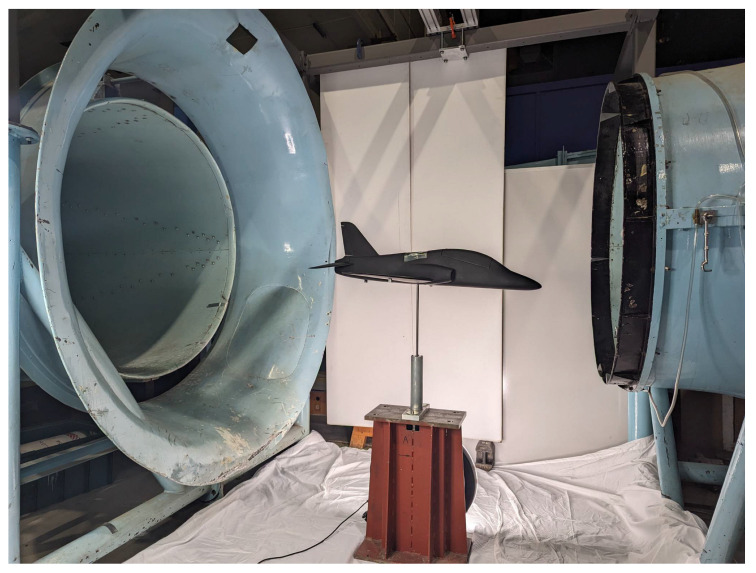
3-DOF aircraft model in DWT.

The preferred choice for conducting free-flying testing is typically a 3-DOF rotational motion rig, enabling force measurement either via a balance positioned at the rig mount or the attachment point of the model. At Cranfield University, the available DWT testing rig features an open-section tunnel with a 1.5 × 1.1 m working section and a maximum speed capability of 40 m/s, facilitating investigation into four degrees of freedom (roll, pitch, yaw, and heave) of motion. This setup allows for a roll and pitch motion range of ±30°, a yaw motion range of 360°, and a maximum translation motion range of 0.75 m in heave [5,6]. Figure 1 depicts the Cranfield University DWT rig, featuring a 1/12 scale model of the BAE Hawk, enabling 3-DOF motion with a bottom rig mount. Another configuration is the DWT testing rig at the University of Bristol, which has a maximum 5-DOF capability by incorporating a 2-DOF gimbal at the pendulum attachment and a 3-DOF gimbal mounted at the model’s centre of gravity [7,8,9]. Another 4-DOF dynamic wind tunnel pitch, roll, yaw, and heave has been developed [10].

On-board instrumentation plays a crucial role in aircraft model testing, with inertial measurement units (IMUs) being a popular choice for measuring rotation rates and displacement accelerations. IMUs, consisting of three gyroscopes and accelerometers mounted in orthogonal axes, provide measurements in all three dimensions. Despite their effectiveness, IMUs exhibit varying bias performances, with micro-electro-mechanical systems (MEMSs) and ring laser gyro (RLG)-based IMUs from Honeywell ranging from 0.25 deg/h to 7 deg/h and 0.25 deg/h to 0.0006 deg/h, respectively [11]. However, high-accuracy IMUs are often expensive and bulky, posing limitations for DWT testing models. Alternatively, low-cost MEMS sensors can be employed for scaled model testing, although with reduced accuracy due to inherent errors such as bias error, repeatability, stability, drift, sensor noise, misalignment, and latency. To mitigate these errors, sensor fusion techniques including, for example, multiple IMUs and model-based filters such as extended Kalman and Sage-Husa adaptive Kalman filters [12] are widely used. On the contrary, off-board sensors like high-speed cameras offer real-time data acquisition capabilities, presenting an advantage in dynamic wind tunnel testing for identifying flight dynamic models. However, their performance can be hindered by factors like lighting and background settings. The continuous advances in computer vision techniques present an opportunity to enhance the accuracy of data obtained in dynamic wind tunnel experiments.

Computer vision techniques for attitude estimation are often reliant on visual markers placed on the model. However, the absence of markers allows for the implementation of the algorithm across diverse aircraft wing configurations, including high, low, and mid-wing designs. This versatility highlights the potential applicability of the computer vision algorithm without markers, making it adaptable for various aircraft models without being limited to specific configurations.

Moreover, the absence of markers broadens the research application to various fields where computer vision is essential, such as air traffic control tower surveillance [13] and marine operations [14]. While traditional applications focus more on target positions rather than attitudes, this research lays a foundation for incorporating attitude estimation in such scenarios. Additionally, as the use of drones expands across sectors like rescue operations, surveillance, and agriculture, the need for drone identification and classification becomes crucial [15]. The findings of this research can potentially be effectively applied in state estimations for drones, as well as for advanced techniques aimed at accurately identifying, tracking, and estimating missile dynamics for military purposes.

In this paper, we propose a method for accurately acquiring dynamic wind tunnel data for a scaled rigid-body aircraft model without visual markers on the model. The method employs a sequential process involving Harris corner detection and Kanade–Lucas–Tomasi tracking. From this, an aircraft skeleton position is obtained. Then, the Euler angles and Euler rates may be estimated to capture the attitude of the aircraft model. Validation of this approach is conducted by simulating a 3D CAD model of a 1/12 sub-scale BAE Hawk fighter aircraft model using MATLAB Simscape. Note that an early version of this work was previously presented in [16] and this paper improves and extends the previous study in several key areas. Quaternions for the attitude estimation are used instead of the direction cosine matrix and this improves the accuracy of the results. Notably, a detailed sensitivity analysis on the use of quaternions is conducted. The analysis identifies first-order changes in the estimated angles resulting from small changes in the identified position of the aircraft axes. Furthermore, this paper adds sensor fusion between on-board and off-board instrumentation by implementing an extended Kalman filter, as suggested in [16].

The contributions of this paper include the following: (i) A sequence of established computer vision techniques is proposed for solving a new problem. These include feature detection, specifically corner detection and feature tracking to obtain an aircraft skeleton. (ii) A customised transformation method from 2D to 3D coordinates tailored for a stereo orthogonal setup is presented. Furthermore, it adapts well-established Euler angles, commonly used in aerospace for attitude estimation, to estimate Euler angles from the aircraft skeleton points by employing quaternions and transforming them to Euler angles. (iii) A sensitivity analysis is performed; this determines first-order changes in the Euler angles resulting from alterations in the positioning of the aircraft skeleton points. This analysis serves to pinpoint the aircraft skeleton points that exert the most significant impact on the Euler angles, enhancing the accuracy and precision of the estimation process. (iv) Finally, by combining these advances, this interdisciplinary research proposes a complete framework for accurate attitude estimation in dynamic wind tunnel applications, with a focus on identifying and mitigating potential sources of error.

The rest of this paper is structured as follows. Section 2 discusses the related work pertinent to the innovation presented in this paper, namely the application of computer vision techniques for estimating the attitude of an aircraft model during dynamic wind tunnel testing. Section 3 discusses attitude estimation using our proposed method. Section 4 details the 3D CAD model simulation. Section 5 discusses the conducted sensitivity analysis. Section 6 elaborates on the extended Kalman filter utilised for sensor fusion, integrating data from both the off-board and on-board sensors. Section 7 discusses the results. Our conclusions are presented in Section 8.

## 2. Related Work

Various object detection techniques utilising laser and self-luminous markers [17], as well as multiple line marks on the sides of train models [18] and markers [19], have been employed for pose measurements in wind tunnels. Additionally, object-tracking techniques, including particle filtering [20], saliency combined particle filtering [21], and background subtraction [22], have been utilised for aerial UAV tracking.

Extensive research has been conducted on using photogrammetry for various applications, including obtaining aerodynamic parameters from wind tunnel experiments. At NASA’s Langley Research Center, a digital photogrammetry technique has been employed to measure model deformation, and sting bending using a single camera [23]. This method allows for the extraction of 2D or 3D spatial measurements, which are then transformed into engineering variables such as attitude and deformation. By leveraging the capabilities of computer vision to perform autonomous algorithms, the accuracy and speed of these measurements are significantly improved. Specifically, computer vision was utilised to monitor model deformation. The primary goal of this work was to enhance the deformation measurement capability, which also holds potential for measuring model attitude.

A binocular vision method using a photogrammetry model based on multi-layer refraction and laser strips has been proposed for measuring the position and attitude of high-speed rolling targets in wind tunnel experiments [24]. Laser-aided vision technology for high-precision pose measurement in wind tunnels has also been suggested for rolling targets, though improvements could include speed measurement and increased precision [17]. A monocular vision method using coloured images achieves accurate positioning through image differencing, accumulation, and marker position estimation, with precision being less than 0.19 mm and 0.18° for the position and angle of pitch and yaw [25].

For supersonic wind tunnels, a pose measurement method employing ultrathin retro-reflection markers and spatial coding achieves high accuracy, with precision values of less than 0.16 mm, 0.132°, and 0.712° for position, pitch, and yaw, respectively [26]. Additionally, an attitude and position measurement system for slender models using two cameras, one positioned to the side and the other looking up the model, achieves a maximal error of less than 0.05° for attitude measurement [27].

A photogrammetry system using two video cameras has been proposed for measuring the attitude of models in hypersonic wind tunnels, achieving an accuracy of 0.1° for pitch, although these results have not been independently validated [28]. Another approach has been suggested using a single camera, offering a flexible position and attitude measurement method [29]. In this method, the focal length and camera orientation can be adjusted for various measurement conditions without needing to recalibrate the camera. This technique can achieve an accuracy of 0.2866 mm for displacement measurements of planar targets and 0.2615° for attitude measurements of planar objects.

A remote sensing system using a motion capture camera for the position measurement of a T-shaped object with visual markers has been proposed [30]. This method has been compared with the commonly used laser displacement sensor (LDS) method, and the results indicate that the motion capture method is equally accurate. Additionally, a pose measurement technique for an aircraft model in a wire-driven parallel suspension system (WDPSS) using monocular vision and visual markers, along with HALCON for data processing, has been suggested [31]. While this method is feasible, further work is needed to improve accuracy and achieve dynamic real-time measurement.

A method using two high-speed industrial cameras and high-contrast targets on an aircraft model surface has been proposed for the angle of attack measurement, with accuracy within 0.01° [32]. This method shows promise for effective measurement in high-speed wind tunnels. Another approach, employing monocular vision and visual markers on the model along with HALCON for data processing, has been suggested, providing accurate results with a relative error of less than 2% compared to three-axis turntable measurements [33]. However, there is room for enhancement in this method.

For spin tests in vertical wind tunnels, a method utilising two CCD cameras and visual markers achieves an accuracy of 0.1° for attitude angle measurement [34]. Another method based on stereo vision, involving the projection of collimated laser beams onto screens, demonstrates promising results with maximum deviations of less than 0.05° for pitch, roll, and yaw angles, whilst validating in a controlled laboratory environment [35].

The only existing literature on estimating aircraft dynamics using computer vision without markers conducted by [36], explores computer vision-based estimation of aircraft dynamics, employing optical flow data captured by a downward-looking camera and processed through an extended Kalman Filter, complemented by Monte Carlo simulations for analysis. In contrast, our research adopts a novel approach utilising 3D CAD simulations to replicate real-world conditions and environments, facilitating the reproduction of real-world experiments, aiding in error identification and setting multiple degrees of freedom for the model. MATLAB Simscape serves as a valuable tool for modelling, simulating, and visualising the CAD model. While [36] utilise Monte Carlo simulations primarily for statistical analysis and uncertainty assessment, our Simscape simulations concentrate on the dynamic behaviour and performance analysis of physical systems, enabling the examination of time-domain response, transient behaviour, steady-state characteristics, and control of physical systems. Simscape proves particularly beneficial for designing and testing control systems and assessing system performance under diverse conditions. Compared to the approach by [36], our method offers a cost-effective alternative before conducting dynamic wind tunnel testing, producing results comparable to real-world experimental outcomes.

## 3. Attitude Estimation

This section elaborates on the methodology employed for attitude estimation of an aircraft model using computer vision, without the need for visual markers on the model. The approach follows a systematic process that involves several key steps:

1. Feature identification: Initially, key features representing the aircraft’s skeleton are identified within image frames extracted from video footage. The video is captured using an orthogonal stereo camera setup, which provides both top and side views of the aircraft. This stereo vision is critical for accurate spatial analysis. Section 3.1 details feature identification and tracking in initial and consecutive frames.
2. Two-dimensional (2D) to 3D transformation: Once the key features are identified, their coordinates in the 2D-pixel coordinates are transformed into 3D coordinates. This transformation leverages the orthogonal views provided by the stereo camera setup to accurately reconstruct the spatial configuration of the identified points. Section 3.2 discusses the theory of the pinhole camera model to transform the 2D pixel coordinates to 3D coordinates. In addition, Section 3.3 details the tailored method for the transformation of 2D pixel coordinates to complete (X, Y, Z) 3D coordinates.
3. Attitude estimation: With the 3D coordinates of the key features obtained, the next step involves estimating the aircraft’s attitude. This is achieved by employing quaternions, a mathematical representation that facilitates smooth and continuous rotation calculations. The Euler angles, which describe the aircraft’s orientation, are derived from these quaternions. Section 3.4 and Section 3.4.1 describe the widely applied Euler angles for aerospace applications and our proposed method for estimating Euler angles from aircraft skeleton points by employing quaternions.

This methodology demonstrates a robust and efficient approach to aircraft attitude estimation using computer vision techniques, eliminating the need for physical markers and leveraging computer vision techniques for accurate spatial analysis.

### 3.1. Features Detection and Tracking

**Figure 2:**
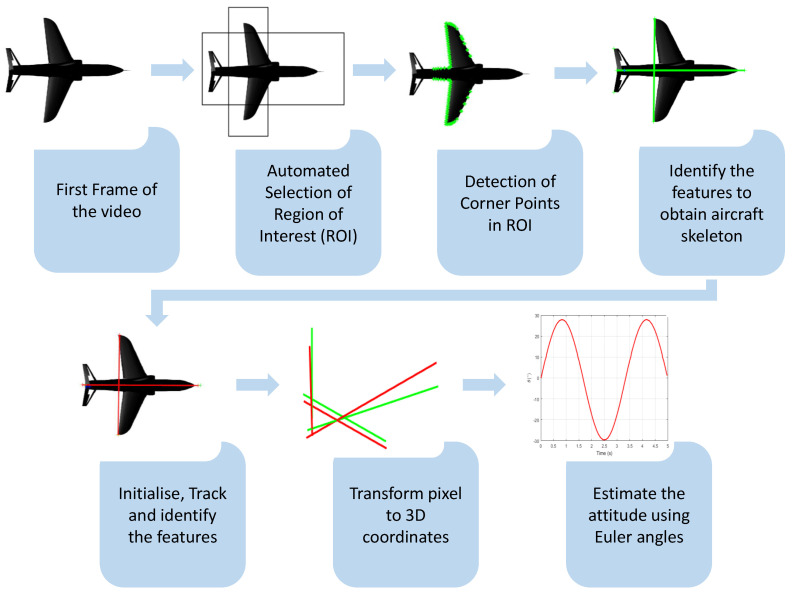
Flow chart showing the process for attitude estimation.

After acquiring the video and extracting initial frames from both top and side views, the region of interest (ROI) is selected, encompassing the wingspan and fuselage length. Subsequently, the focus shifts to detecting corner points within the image. Harris corner and edge detection, as outlined by Harris [37], is a widely recognised approach for corner detection. This method is crucially employed in the feature detection stage of the process as shown in Figure 2 to identify corner points on the aircraft model. The corner points are obtained in these ROIs using the MATLAB Computer Vision Toolbox detectHarrisFeatures function. The details and methodology of Harris corner detection can be found in [37].

The aircraft skeleton is defined by key corner points, including the nose tip, midpoint of the horizontal stabiliser, wingtips, and vertical stabiliser tip. The nose tip and midpoint of the horizontal stabiliser represent the fuselage length along the X-axis of the aircraft. The wingtips define the wingspan along the Y-axis, while the vertical stabiliser tip and an intersecting point from this tip to the fuselage length in the side view define the Z-axis.

These corner points, which include fuselage length and wingspan, are visually identifiable in the top view, while the fuselage length and vertical stabiliser length are easily seen in the side view. The remaining corner points are identified using aircraft geometry and the knowledge of the setup of the 1-DOF test as described in Section 4.

The corner points in the initial frame are used to track the feature point tracking stage shown in Figure 2. The methodology adopted in this research involves the application of the Kanade–Lucas–Tomasi [38,39] tracking technique to strategically monitor selected points within the initial frame, which serve as pivotal landmarks on the model aircraft skeleton. These key points include wing tip points, the nose tip in the top view, and both the tail tip and nose tip in the side view. Subsequent frames are then analysed to identify the remaining corner points of the aircraft skeleton by implementing the Harris corner detection through a synthesis of aircraft geometry and insights gained from the experimental test procedures conducted as described in Section 4. The operation of the Kanade–Lucas–Tomasi tracking algorithm, which estimates the displacement between an initialised point and the next frame to track the point across frames, is detailed in [38,39]. This approach ensures precise and comprehensive tracking of the model aircraft movement, facilitating a thorough analysis of its behaviour and performance characteristics.

### 3.2. Camera Parameters

**Figure 3:**
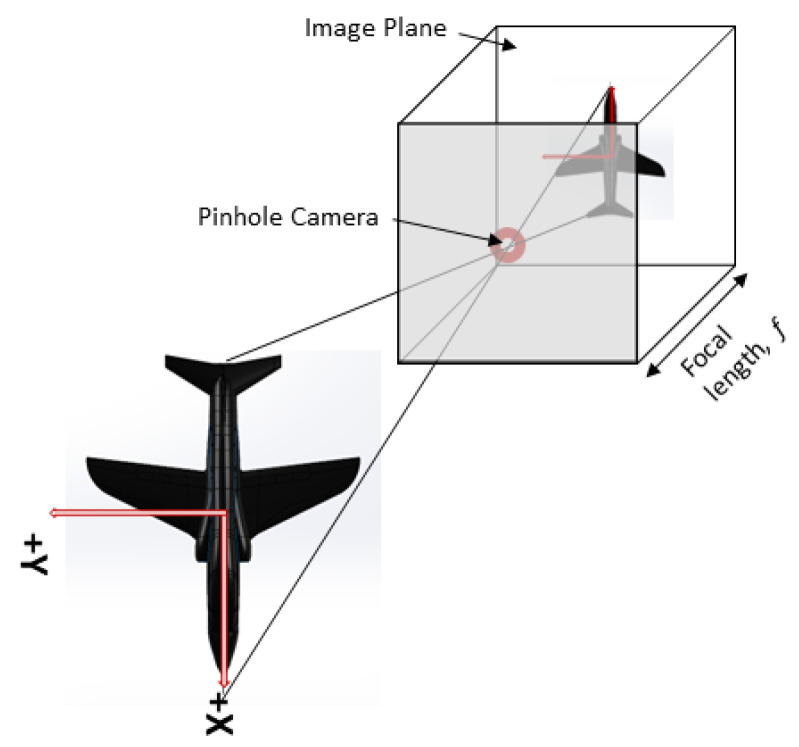
Pinhole camera [16] (reprinted with permission from the American Institute of Aeronautics and Astronautics, Inc.).

The subsequent section delves into the camera parameters based on a widely known pinhole camera model. The objective is to detect the location of corner points within a 3D space based on their corresponding image points, which result from projecting the 3D points onto a plane. This is illustrated in Figure 3. The transformation is described in detail here and serves as the penultimate stage within the process outlined in Figure 2.

#### 3.2.1. Extrinsic Parameters of the Simscape Model

The rotation matrix, $R_{T}$, of body coordinates $\left(X_{b},Y_{b},Z_{b}\right)$ to camera coordinates $\left(X_{c},Y_{c},Z_{c}\right)$ in Simscape for a top view is given by the following:

(1) $$R_{T}=\left[\begin{array}{ccc} 1 & 0 & 0\\ 0 & 1 & 0\\ 0 & 0 & 1 \end{array}\right].$$

Similarly, the rotation matrix, $R_{S}$, of body coordinates to camera coordinates in Simscape for a side view is as follows:

(2) $$R_{S}=\left[\begin{array}{ccc} 1 & 0 & 0\\ 0 & 0 & -1\\ 0 & 1 & 0 \end{array}\right].$$

The translation matrices for both the views are as follows:

(3) $$\begin{array}{cc} t_{T} & =\left[\begin{array}{ccc} 0 & 0 & -50\;\mathrm{cm} \end{array}\right] \end{array}$$

(4) $$\begin{array}{cc} t_{S} & =\left[\begin{array}{ccc} 0 & 50\;\mathrm{cm} & 0 \end{array}\right]. \end{array}$$

#### 3.2.2. Transformation from 3D to 2D

The extrinsic parameters of rotation and translation are multiplied with a 3D point to obtain the point in the camera coordinate system by the following:

(5) $$\left[\begin{array}{c} X_{c}\\ Y_{c}\\ Z_{c} \end{array}\right]=\left[\begin{array}{cc} R & t \end{array}\right]\left[\begin{array}{c} X\\ Y\\ Z\\ 1 \end{array}\right].$$

where *R* is a rotation matrix of body coordinates to camera coordinates, and *t* is a translation vector. Multiplying this with the camera’s intrinsic parameters of focal length, *f*, and principal points, $c_{x}$, $c_{y}$, we obtain the 3D point in the pixel coordinates given by the following:

(6) $$\left[\begin{array}{c} u^{\prime }\\ v^{\prime }\\ w^{\prime } \end{array}\right]=\left[\begin{array}{ccc} f & 0 & c_{x}\\ 0 & f & c_{y}\\ 0 & 0 & 1 \end{array}\right]\left[\begin{array}{c} X_{c}\\ Y_{c}\\ Z_{c} \end{array}\right].$$

The 2D-pixel coordinates are then obtained by the following:

(7) $$u=\frac{u^{\prime }}{w^{\prime }},\;v=\frac{v^{\prime }}{w^{\prime }}.$$

The camera projection parameters are chosen for this simulation as follows. The principal point is positioned at the centre of gravity of the model in the top and side views. By aligning the principal point with the centre of gravity of the aircraft model, a common reference point is established for both the top and side views. This alignment simplifies the geometric transformations required for 3D reconstruction, reducing parallax errors and enhancing the accuracy of depth estimation. Moreover, this configuration streamlines mathematical modelling and camera calibration processes. It also improves the interpretability and visualisation of the aircraft model structure and dimensions, facilitating more precise and consistent measurements. In addition, the focal length is set to 1, as it does not affect the camera projection matrix in this simulation context. This approach effectively removes any additional complexity that could arise from varying focal lengths or scaling factors, thus streamlining the validation process.

#### 3.2.3. Transformation from 2D to 3D

Transforming a corner point on a 2D plane (identified in Section 3.1) to a 3D point is performed by the following method. The pixel coordinates (u,v) obtained from an image are transformed into image coordinates by the following:

(8) $$\begin{array}{cc} X_{I} & =d_{px}\left(u-c_{x}\right), \end{array}$$

(9) $$\begin{array}{cc} Y_{I} & =d_{py}\left(v-c_{y}\right), \end{array}$$

where $c_{x},c_{y}$ are the principal points and $\left(d_{px},d_{py}\right)$ are the metres/pixel scaling pair chosen to be to (1,1) for simplicity. Since all the points lie on the image plane, the camera coordinates are obtained by the following:

(10) $$\left[\begin{array}{c} X_{c}\\ Y_{c}\\ Z_{c} \end{array}\right]=\left[\begin{array}{c} X_{I}\\ Y_{I}\\ f \end{array}\right].$$

The camera coordinates are transformed into 3D coordinates by the following:

(11) $$\left[\begin{array}{c} X\\ Y\\ Z\\ 1 \end{array}\right]=\left[\begin{array}{cc} R^{T} & t^{T} \end{array}\right]\left[\begin{array}{c} X_{c}\\ Y_{c}\\ Z_{c} \end{array}\right].$$

where *R* is a rotation matrix of body coordinates to camera coordinates, *t* is a translation vector, and ${\left(\cdot \right)}^{T}$ denotes the transpose. These transformations are applied to both the top and side views.

#### 3.2.4. Camera Pose Estimation and Triangulation

The relative pose of the side camera to the top camera can be estimated by using the knowledge of the extrinsic parameters of both cameras by the following:

(12) $$\begin{array}{cc} R_{R} & =R_{T}^{\prime }R_{S} \end{array}$$

(13) $$\begin{array}{cc} t_{R} & =t_{S}-t_{T} \end{array}$$

Using the above-known parameters and triangulating 3D points by the function triangulate based on direct linear transformation [40] induces errors in identifying the corresponding 3D points due to the sensitivity limitation to noise.

### 3.3. 3D Aircraft Model Skeleton

**Figure 4:**
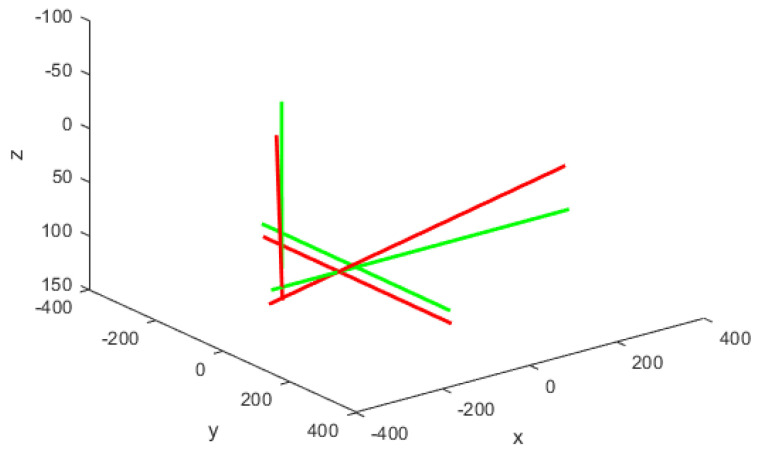
Aircraft skeleton in 3D. Initial frame shown in green, next frame shown in red.

The transformation of identified 2D pixel coordinates from the top and side views as described in Section 3.1 into 3D coordinates is achieved using the respective camera projection matrices as described in Section 3.2. This process yields accurate (X, Y) 3D coordinates from the top view and (X, Z) 3D coordinates from the side view. The complete 3D coordinates of the aircraft skeleton are then obtained by averaging the shared X-coordinate from both views, using the Y-coordinate from the top view and the Z-coordinate from the side view. Integrating corner points from both camera perspectives by utilising this tailored method for orthogonal stereo setup yields the complete aircraft skeleton, as depicted in Figure 4.

### 3.4. Euler Angles

The final stage of the process shown in Figure 2 is to extract the Euler or Tait–Bryan angles (roll, pitch, yaw). The aircraft model is given the standard right-hand set body axes used for aircraft dynamics [16] with the *X*-axis pointing forward, the *Y*-axis pointing starboard, and the *Z*-axis pointing downward. The rotation matrices for roll, pitch, and yaw are given next. The rotation matrix for a roll rotation around the *X*-axis through the angle ϕ is given by the following:

(14) $$R_{X}=\left[\begin{array}{ccc} 1 & 0 & 0\\ 0 & \cos\phi & \sin\phi \\ 0 & -\sin\phi & \cos\phi \end{array}\right]$$

The rotation matrix around the new *Y*-axis is a pitch rotation through the angle θ and is given by the following:

(15) $$R_{Y}=\left[\begin{array}{ccc} \cos\theta & 0 & -\sin\theta \\ 0 & 1 & 0\\ \sin\theta & 0 & \cos\theta \end{array}\right]$$

The final rotation is around the Earth frame *Z*-axis through a yawn angle ψ with a rotation matrix

(16) $$R_{Z}=\left[\begin{array}{ccc} \cos\psi & \sin\psi & 0\\ -\sin\psi & \cos\psi & 0\\ 0 & 0 & 1 \end{array}\right]$$

The rotation matrix (known as the direction cosine matrix) around the *X*-$Y^{\prime }$-$Z^{\prime \prime }$ axes through the angles ϕ, θ, ψ is given by the following:

(17) $$R=\left[\begin{array}{ccc} \cos\theta \;\cos\psi & \cos\theta \;\sin\psi & -\sin\theta \\ \sin\phi \;\sin\theta \;\cos\psi -\cos\phi \;\sin\psi & \sin\phi \;\sin\theta \;\sin\psi +\cos\phi \;\cos\psi & \sin\phi \;\cos\theta \\ \cos\phi \;\sin\theta \;\cos\psi +\sin\phi \;\sin\psi & \cos\phi \;\sin\theta \;\sin\psi -\sin\phi \;\cos\psi & \cos\phi \;\cos\theta \end{array}\right]$$

The Euler angles can be extracted from the DCM, (*R*), and are given by the following:

(18) $$\begin{array}{cc} \theta & =-\arcsin\left(R_{13}\right), \end{array}$$

(19) $$\begin{array}{cc} \phi & =\arctan\left(\frac{R_{23}}{R_{33}}\right), \end{array}$$

(20) $$\begin{array}{cc} \psi & =\arctan\left(\frac{R_{12}}{R_{11}}\right). \end{array}$$

The Euler rates are computed to derive dynamic measurements for attitude estimation. These rates provide crucial insights into the aircraft dynamics, aiding in the accurate determination of its orientation over time. The Euler rates ($\dot{\phi },\dot{\theta },\dot{\psi }$) from the body angular rates (p,q,r) are given by the following:

(21) $$\left[\begin{array}{c} \dot{\phi }\\ \dot{\theta }\\ \dot{\psi } \end{array}\right]=\left[\begin{array}{ccc} 1 & \sin\phi \;\tan\theta & \cos\phi \;\tan\theta \\ 0 & \cos\phi & -\sin\phi \\ 0 & \sin\phi \;\sec\theta & \cos\phi \;\sec\theta \end{array}\right]\left[\begin{array}{c} p\\ q\\ r \end{array}\right]$$

Since the body angular rate *p* is unknown, the Euler rate $\left(\dot{\phi }\right)$ is obtained by backward difference numerical differentiation as follows:

(22) $$\dot{\phi }=\frac{\phi (t)-\phi (t-1)}{\Delta t}.$$

Although this method causes a time shift, it is a feasible solution for implementation in a real-time system. The angular rates $\dot{\theta }$ and $\dot{\psi }$ are similarly obtained.

#### 3.4.1. Euler Angles and Rates from Aircraft Skeleton

Let $P_{0}$ represent a matrix containing the initial skeleton points on the aircraft model before an Euler rotation in 3D coordinates and $P_{3}$ represent the matrix containing corresponding points on the aircraft model after an Euler rotation in 3D coordinates. These are points obtained as described in Section 3.3. The matrix $P_{3}$ obtained can have the expanded equation as follows:

(23) $$P_{3}=\left[\begin{array}{cccc} {X_{3}}_{\left(1,1\right)} & {X_{3}}_{\left(1,2\right)} & \cdots & {X_{3}}_{\left(1,6\right)}\\ {Y_{3}}_{\left(2,1\right)} & {Y_{3}}_{\left(2,2\right)} & \cdots & {Y_{3}}_{\left(2,6\right)}\\ {Z_{3}}_{\left(3,1\right)} & {Z_{3}}_{\left(3,2\right)} & \cdots & {Z_{3}}_{\left(3,6\right)} \end{array}\right]$$

The X-axis vector of the aircraft model is at ${P_{3}}_{\left(i,j\right)}$, where i=1,2,3 and j=1,2 are the rows and columns of the matrix, respectively. The resultant vector and the normalised vector are obtained by the following:

(24) $$X_{\left(3\right)}=\left[\begin{array}{c} {X_{3}}_{\left(1,2\right)}-{X_{3}}_{\left(1,1\right)}\\ {Y_{3}}_{\left(2,2\right)}-{Y_{3}}_{\left(2,1\right)}\\ {Z_{3}}_{\left(3,2\right)}-{Z_{3}}_{\left(3,1\right)} \end{array}\right]$$

To obtain the X-axis vector with unity magnitude and without changing its direction, the unit vectors (i, j, and k) of the vector can be obtained by normalising the vector as follows:

(25) $${\hat{X}}_{\left(3\right)}=\frac{X_{\left(3\right)}}{∥X_{\left(3\right)}∥}$$

where $∥X_{\left(3\right)}∥$ denotes the Euclidean norm of $X_{\left(3\right)}$.

Similarly, the Y-axis vector of the aircraft model is at ${P_{3}}_{\left(i,j\right)}$, where i=1,2,3 and j=3,4 are the rows and columns of the matrix, respectively. The resultant vector and the normalised vector are obtained by the following:

(26) $$\begin{array}{cc} Y_{\left(3\right)} & =\left[\begin{array}{c} {X_{3}}_{\left(1,4\right)}-{X_{3}}_{\left(1,3\right)}\\ {Y_{3}}_{\left(2,4\right)}-{Y_{3}}_{\left(2,3\right)}\\ {Z_{3}}_{\left(3,4\right)}-{Z_{3}}_{\left(3,3\right)} \end{array}\right], \end{array}$$

(27) $$\begin{array}{cc} {\hat{Y}}_{\left(3\right)} & =\frac{Y_{\left(3\right)}}{∥Y_{\left(3\right)}∥}. \end{array}$$

Finally, the Z-axis vector of the aircraft model is at ${P_{3}}_{\left(i,j\right)}$, where i=1,2,3 and j=5,6 are the rows and columns of the matrix, respectively. The resultant vector and the normalised vector are obtained by the following:

(28) $$\begin{array}{cc} Z_{\left(3\right)} & =\left[\begin{array}{c} {X_{3}}_{\left(1,6\right)}-{X_{3}}_{\left(1,5\right)}\\ {Y_{3}}_{\left(2,6\right)}-{Y_{3}}_{\left(2,5\right)}\\ {Z_{3}}_{\left(3,6\right)}-{Z_{3}}_{\left(3,5\right)} \end{array}\right], \end{array}$$

(29) $$\begin{array}{cc} {\hat{Z}}_{\left(3\right)} & =\frac{Z_{\left(3\right)}}{∥Z_{\left(3\right)}∥}. \end{array}$$

The rotation matrix for the orientation is given by the following:

(30) $${Q^{T}}_{\left(3\right)}=\left[\begin{array}{ccc} {\hat{X}}_{\left(3\right)}, & {\hat{Y}}_{\left(3\right)}, & {\hat{Z}}_{\left(3\right)} \end{array}\right]$$

The matrix is used to extract the quaternion components (${q_{\left(3\right)}}_{w}$, ${q_{\left(3\right)}}_{x}$, ${q_{\left(3\right)}}_{y}$, ${q_{\left(3\right)}}_{z}$) of ${Q^{T}}_{\left(3\right)}$ as follows:

(31) $$\begin{array}{cc} {q_{\left(3\right)}}_{w} & =\frac{1}{2}\sqrt{\left(1+\frac{{X_{3}}_{\left(1,2\right)}-{X_{3}}_{\left(1,1\right)}}{∥X_{\left(3\right)}∥}+\frac{{Y_{3}}_{\left(2,4\right)}-{Y_{3}}_{\left(2,3\right)}}{∥Y_{\left(3\right)}∥}+\frac{{Z_{3}}_{\left(3,6\right)}-{Z_{3}}_{\left(3,5\right)}}{∥Z_{\left(3\right)}∥}\right)} \end{array}$$

(32) $$\begin{array}{cc} {q_{\left(3\right)}}_{x} & =\frac{1}{4{q_{\left(3\right)}}_{w}}\left(\frac{{Y_{3}}_{\left(2,6\right)}-{Y_{3}}_{\left(2,5\right)}}{∥Z_{\left(3\right)}∥}-\frac{{Z_{3}}_{\left(3,4\right)}-{Z_{3}}_{\left(3,3\right)}}{∥Y_{\left(3\right)}∥}\right) \end{array}$$

(33) $$\begin{array}{cc} {q_{\left(3\right)}}_{y} & =\frac{1}{4{q_{\left(3\right)}}_{w}}\left(\frac{{Z_{3}}_{\left(3,2\right)}-{Z_{3}}_{\left(3,1\right)}}{∥X_{\left(3\right)}∥}-\frac{{X_{3}}_{\left(1,6\right)}-{X_{3}}_{\left(1,5\right)}}{∥Z_{\left(3\right)}∥}\right) \end{array}$$

(34) $$\begin{array}{cc} {q_{\left(3\right)}}_{z} & =\frac{1}{4{q_{\left(3\right)}}_{w}}\left(\frac{{X_{3}}_{\left(1,4\right)}-{X_{3}}_{\left(1,3\right)}}{∥Y_{\left(3\right)}∥}-\frac{{Y_{3}}_{\left(2,2\right)}-{Y_{3}}_{\left(2,1\right)}}{∥X_{\left(3\right)}∥}\right) \end{array}$$

Since the aircraft model is trimmed in the initial frame and has no rotation, the initial quaternion matrix obtained is an identity matrix. Therefore, the quaternion matrix and the quaternion components are as follows:

(35) $${Q^{T}}_{\left(0\right)}=\left[\begin{array}{ccc} 1 & 0 & 0\\ 0 & 1 & 0\\ 0 & 0 & 1 \end{array}\right]$$

(36) $$\begin{array}{c} {q_{\left(0\right)}}_{w}=1,\;{q_{\left(0\right)}}_{x}=0,\;{q_{\left(0\right)}}_{y}=0,\;\mathrm{and}\;{q_{\left(0\right)}}_{z}=0. \end{array}$$

The relative orientation between the two frames is obtained from the quaternions by the following:

$$\begin{array}{c} q_{\left(r\right)}=q_{\left(3\right)}q_{\left(0\right)}^{-1} \end{array}$$

where $q_{\left(.\right)}$ is the quaternion, including the scalar and the unit vector components. The inverse of a quaternion (*q*) is equal to the complex conjugate $\left(q^{*}\right)$ of the quaternion when the quaternion is normalised; that is, ${\left|q\right|}^{2}=q_{w}^{2}+q_{x}^{2}+q_{y}^{2}+q_{z}^{2}=1$. Therefore, $q_{\left(0\right)}^{-1}=q_{\left(0\right)}^{*}$, and the equation is modified as follows:

$$\begin{array}{c} q_{\left(r\right)}=q_{\left(3\right)}q_{\left(0\right)}^{*} \end{array}$$

where the complex conjugate $q_{\left(0\right)}^{*}={q_{\left(0\right)}}_{w}-i{q_{\left(0\right)}}_{x}-j{q_{\left(0\right)}}_{y}-k{q_{\left(0\right)}}_{z}$. By using the quaternion product rule, the relative orientation can be rewritten as follows:

(37) $$q_{\left(r\right)}={q_{\left(3\right)}}_{w}+q_{\left(3\right)}=q_{\left(3\right)}$$

where ${q_{\left(3\right)}}_{w}$ is the scalar component and $q_{\left(3\right)}$ is the unit vector component of the quaternion. The quaternion components (${q_{\left(3\right)}}_{w}$, ${q_{\left(3\right)}}_{x}$, ${q_{\left(3\right)}}_{y}$, and ${q_{\left(3\right)}}_{z}$) are used to obtain the Euler angles (ϕ, θ, and ψ) using the following equations:

(38) $$\begin{array}{cc} \phi & =\arctan\left(\frac{{{Q^{T}}_{\left(3\right)}}_{\left(2,3\right)}}{{{Q^{T}}_{\left(3\right)}}_{\left(3,3\right)}}\right)=\arctan\left(\frac{2{q_{\left(3\right)}}_{y}{q_{\left(3\right)}}_{z}+2{q_{\left(3\right)}}_{w}{q_{\left(3\right)}}_{x}}{{q_{\left(3\right)}}_{w}^{2}-{q_{\left(3\right)}}_{x}^{2}-{q_{\left(3\right)}}_{y}^{2}+{q_{\left(3\right)}}_{z}^{2}}\right) \end{array}$$

(39) $$\begin{array}{cc} \theta & =-\arcsin\left({{Q^{T}}_{\left(3\right)}}_{\left(1,3\right)}\right)=-\arcsin\left(\frac{2{q_{\left(3\right)}}_{x}{q_{\left(3\right)}}_{z}-2{q_{\left(3\right)}}_{w}{q_{\left(3\right)}}_{y}}{{q_{\left(3\right)}}_{w}^{2}+{q_{\left(3\right)}}_{x}^{2}+{q_{\left(3\right)}}_{y}^{2}+{q_{\left(3\right)}}_{z}^{2}}\right) \end{array}$$

(40) $$\begin{array}{cc} \psi & =\arctan\left(\frac{{{Q^{T}}_{\left(3\right)}}_{\left(1,2\right)}}{{{Q^{T}}_{\left(3\right)}}_{\left(1,1\right)}}\right)=\arctan\left(\frac{2{q_{\left(3\right)}}_{x}{q_{\left(3\right)}}_{y}+2{q_{\left(3\right)}}_{w}{q_{\left(3\right)}}_{z}}{{q_{\left(3\right)}}_{w}^{2}+{q_{\left(3\right)}}_{x}^{2}-{q_{\left(3\right)}}_{y}^{2}-{q_{\left(3\right)}}_{z}^{2}}\right) \end{array}$$

## 4. 3D CAD Simulation

A 3D CAD rendition of a 1/12 scaled BAE Hawk is crafted using SolidWorks (Student version 2021-22).This model is then imported into MATLAB Simscape (version R2023a) for modelling and simulating the aircraft motion. Within the Simscape environment, a model is constructed featuring gimbal joints to facilitate the Euler angle transformation of the CAD model, alongside a transform sensor to extract the rotation matrix. Subsequently, the angles ϕ, θ, and ψ are applied to the Simscape model.

The presented experiment tests just a single degree-of-freedom, whereby the pitch angle undergoes sinusoidal variation and the roll and yaw are fixed at zero. This experiment demonstrates the method using a longitudinal motion rig; additional work is ongoing to develop a novel 3-DOF rig capable of testing both the short-period and phugoid modes [41,42].

**Figure 5:**
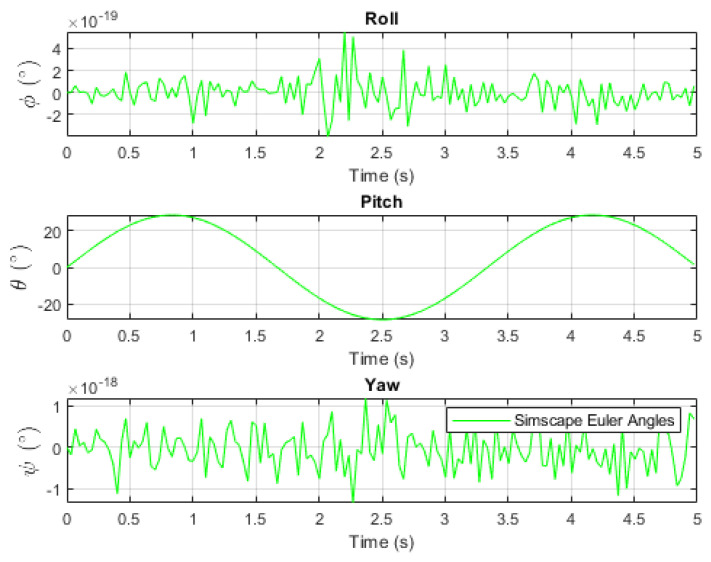
Simscape Euler angles [16].

**Figure 6:**
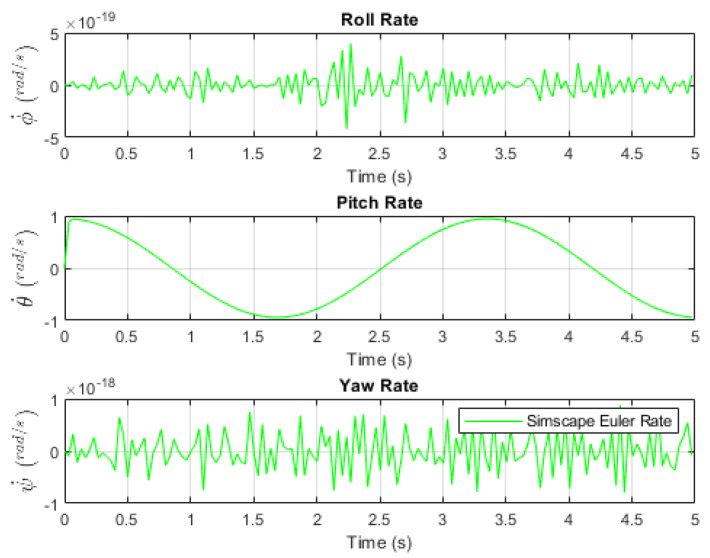
Simscape Euler rates [16].

Figure 5 and Figure 6 depict the applied pitch angle and rate. It is worth noting the presence of numerical rounding noise in the roll and yaw angles.

Realistic data from an on-board sensor comprising an IMU and potentiometer gimbal is generated to facilitate sensor fusion. This process is briefly outlined here. After acquiring the Euler rates as described in Equation (22), we can determine (p,q,r) by taking the inverse of Equation (21). Gyroscope measurements of body angular rates are typically subject to noise. However, in this simulation, ideal (noise-free) measurements of body angular rates (p,q,r) are utilised as ground truth data to validate the state estimates obtained from the EKF. Similarly, the ideal measurements of an accelerometer can be derived using the rotation matrix *R* as mentioned in Equation (17) from the Simscape transform sensor, as outlined below:

$$\left[\begin{array}{c} acc_{x}\\ acc_{y}\\ acc_{z} \end{array}\right]=R^{T}\left[\begin{array}{c} 0\\ 0\\ 9.81 \end{array}\right]$$

To introduce noise and uncertainty into the gyroscope, accelerometer, and potentiometer measurements, zero-mean Gaussian noise is added as unbiased noise, along with a linear drift rate to the gyroscope measurements. The unbiased noise is generated using the normrnd function. The linear drift rate represents the accumulated bias drift over the test duration.

The generated noisy data are filtered through a low-pass filter, effectively reducing noise by attenuating high-frequency components while preserving low-frequency signals. This process also serves to smooth out fluctuations or jitter arising from mechanical imperfections, resulting in a stable and consistent output signal.

## 5. Sensitivity Analysis

A sensitivity analysis is conducted to approximate the change in functions due to small perturbations in its input variables. This is done by utilising Taylor’s first-order series. The analysis is performed by calculating the Jacobian matrices $\partial \phi /\partial P_{3}$, $\partial \theta /\partial P_{3}$, $\partial \psi /\partial P_{3}$ with respect to each of the 6 final frame world coordinates (X,Y,Z) of the aircraft skeleton as obtained in Section 3.3. Euler angle Equations (38)–(40) can be rewritten for the sensitivity analysis as follows:

(41) $$\begin{array}{cc} \phi & =\arctan\left(\frac{2\;\mathrm{num}\;y\;\mathrm{num}\;z+8\;\mathrm{num}\;x\;{{q_{\left(3\right)}}_{w}}^{2}}{16\;{{q_{\left(3\right)}}_{w}}^{4}-{\mathrm{num}\;x}^{2}-{\mathrm{num}\;y}^{2}+{\mathrm{num}\;z}^{2}}\right) \end{array}$$

(42) $$\begin{array}{cc} \theta & =-\arcsin\left(\frac{2\;\mathrm{num}\;x\;\mathrm{num}\;z-\mathrm{num}\;y\;8{{q_{\left(3\right)}}_{w}}^{2}}{16{{q_{\left(3\right)}}_{w}}^{4}+{\mathrm{num}\;x}^{2}+{\mathrm{num}\;y}^{2}+{\mathrm{num}\;z}^{2}}\right) \end{array}$$

(43) $$\begin{array}{cc} \psi & =\arctan\left(\frac{2\;\mathrm{num}\;x\;\mathrm{num}\;y+8\;\mathrm{num}\;z\;{{q_{\left(3\right)}}_{w}}^{2}}{16\;{{q_{\left(3\right)}}_{w}}^{4}+{\mathrm{num}\;x}^{2}-{\mathrm{num}\;y}^{2}-{\mathrm{num}\;z}^{2}}\right) \end{array}$$

where numx, numy, and numz are the numerators of the quaternion components ${q_{\left(3\right)}}_{x}$, ${q_{\left(3\right)}}_{y}$, and ${q_{\left(3\right)}}_{z}$ given in Equations (32)–(34). The Taylor series functions can be given as follows:

(44) $$\begin{array}{c} \phi \left(P_{3}+\delta P_{3}\right)\approx \phi \left(P_{3}\right)+\delta P_{3}\left(\frac{\partial \phi }{\partial P_{3}}\right) \end{array}$$

(45) $$\begin{array}{c} \theta \left(P_{3}+\delta P_{3}\right)\approx \theta \left(P_{3}\right)+\delta P_{3}\left(\frac{\partial \theta }{\partial P_{3}}\right) \end{array}$$

(46) $$\begin{array}{c} \psi \left(P_{3}+\delta P_{3}\right)\approx \psi \left(P_{3}\right)+\delta P_{3}\left(\frac{\partial \psi }{\partial P_{3}}\right) \end{array}$$

## 6. Extended Kalman Filter

An extended Kalman filter is employed for sensor fusion of accelerometer, gyroscope, potentiometer gimbal, and vision data. The EKF enables a comprehensive understanding of the system’s behaviour. Through a process of prediction and correction, the filter refines its estimates over time, providing a fused representation of the underlying system state. This approach not only enhances accuracy but also improves robustness in scenarios where individual sensors may be prone to noise, biases, or data loss. A standard approach is used as follows:

(47) $$\begin{array}{cc} {\hat{x}}_{\left(k|k-1\right)} & =f\left({\hat{x}}_{\left(k-1|k-1\right)},u_{\left(k-1\right)}\right)+w_{k} \end{array}$$

(48) $$\begin{array}{cc} P_{\left(k|k-1\right)} & =F_{k}P_{\left(k-1|k-1\right)}F_{k}^{T}+Q_{\left(k-1\right)} \end{array}$$

(49) $$\begin{array}{cc} z_{k} & =h\left({\hat{x}}_{\left(k|k-1\right)}\right)+v_{k} \end{array}$$

(50) $$\begin{array}{cc} {\tilde{y}}_{k} & =z_{k}-h\left({\hat{x}}_{\left(k|k-1\right)}\right) \end{array}$$

(51) $$\begin{array}{cc} S_{k} & =H_{k}P_{\left(k|k-1\right)}H_{k}^{T}+R_{k} \end{array}$$

(52) $$\begin{array}{cc} K_{k} & =P_{\left(k|k-1\right)}H_{k}^{T}S_{k}^{-1} \end{array}$$

(53) $$\begin{array}{cc} {\hat{x}}_{\left(k|k\right)} & ={\hat{x}}_{\left(k|k-1\right)}+K_{k}{\tilde{y}}_{k} \end{array}$$

(54) $$\begin{array}{cc} P_{\left(k|k\right)} & =\left(I-K_{k}H_{k}\right)P_{\left(k|k-1\right)} \end{array}$$

where

(55) $$w_{k}∼N(0,Q(k))$$

(56) $$v_{k}∼N(0,R(k))$$

the state transition matrix is given by the following:

(57) $$F_{k}=\frac{\partial f}{\partial x}{|}_{{\hat{x}}_{\left(k-1|k-1\right)},u_{\left(k-1\right)}}$$

and the observation transition matrix is given by the following:

(58) $$H_{k}=\frac{\partial h}{\partial x}{|}_{{\hat{x}}_{\left(k|k-1\right)}}$$

Equations (47), (48) and (57) represent the discrete-time predicted state estimate, covariance estimate, and the state transition matrix. Equations (49)–(54) and (58) represent the measurement model, innovation or measurement residual, innovation covariance, near-optimal Kalman gain, updated state estimate, updated covariance estimate, and observation transition matrix, respectively. A loosely coupled EKF is implemented as this reduces the computational complexity. The state vector (x) is the Euler angles and the biases of the gyroscope and is described by the vector, as follows:

(59) $$x={\left[\begin{array}{cccccc} \phi & \theta & \psi & {\mathrm{bias}}_{x} & {\mathrm{bias}}_{y} & {\mathrm{bias}}_{z} \end{array}\right]}^{T}$$

The state control vector (u) is the angular velocity from the gyroscope and is given as follows:

(60) $$u={\left[\begin{array}{ccc} p & q & r \end{array}\right]}^{T}$$

Given the state control vector, the Euler rates can be obtained by modifying (21) as follows:

(61) $$\left[\begin{array}{c} \dot{\phi }\\ \dot{\theta }\\ \dot{\psi } \end{array}\right]=\left[\begin{array}{ccc} 1 & \sin\phi \;\tan\theta & \cos\phi \;\tan\theta \\ 0 & \cos\phi & -\sin\phi \\ 0 & \sin\phi \;\sec\theta & \cos\phi \;\sec\theta \end{array}\right]\left[\begin{array}{c} p-{\mathrm{bias}}_{x}\\ q-{\mathrm{bias}}_{y}\\ r-{\mathrm{bias}}_{z} \end{array}\right]$$

The predicted state estimate (47) is obtained as follows:

(62) $${\hat{x}}_{\left(k|k-1\right)}={\hat{x}}_{\left(k-1|k-1\right)}+\left[\begin{array}{c} \dot{\phi }\\ \dot{\theta }\\ \dot{\psi }\\ 0\\ 0\\ 0 \end{array}\right]\Delta t$$

The state transition matrix given in (57) is obtained as follows:

(63) $${F_{k}}_{\left(:,1\right)}=\left[\begin{array}{c} 1-\Delta t(\cos\phi \;\tan\theta \;\left({\mathrm{bias}}_{y}-q\right)-\sin\phi \;\tan\theta \left({\mathrm{bias}}_{z}-r\right))\\ \Delta t(\cos\phi \left({\mathrm{bias}}_{z}-r\right)+\sin\phi \left({\mathrm{bias}}_{y}-q\right))\\ \Delta t(\left(\sin\phi \left({\mathrm{bias}}_{z}-r\right)\right)/\cos\theta -\left(\cos\phi \left({\mathrm{bias}}_{y}-q\right)\right)/\cos\theta )\\ 0\\ 0\\ 0 \end{array}\right]$$

(64) $${F_{k}}_{\left(:,2\right)}=\left[\begin{array}{c} -\Delta t(\cos\phi \left({\mathrm{bias}}_{z}-r\right)\left(\tan\theta^{2}+1\right)+\sin\phi \left({\mathrm{bias}}_{y}-q\right)\left(\tan\theta^{2}+1\right))\\ 1\\ -\Delta t(\left(\cos\phi \sin\theta \left({\mathrm{bias}}_{z}-r\right)\right)/\cos\theta^{2}+\left(\sin\phi \sin\theta \left({\mathrm{bias}}_{y}-q\right)\right)/\cos\theta^{2})\\ 0\\ 0\\ 0 \end{array}\right]$$

(65) $${F_{k}}_{\left(:,(3:6)\right)}=\left[\begin{array}{cccc} 0 & -\Delta t & -\Delta t\sin\phi \tan\theta & -\Delta t\cos\phi \tan\theta \\ 0 & 0 & -\Delta t\cos\phi & \Delta t\sin\phi \\ 1 & 0 & -(\Delta t\sin\phi )/\cos\theta & -(\Delta t\cos\phi )/\cos\theta \\ 0 & 1 & 0 & 0\\ 0 & 0 & 1 & 0\\ 0 & 0 & 0 & 1 \end{array}\right]$$

The measurement model for sensor fusion is obtained as follows:

(66) $$z_{k}={\left[\begin{array}{ccccccc} {\mathrm{acc}}_{x} & {\mathrm{acc}}_{y} & {\mathrm{acc}}_{z} & \theta_{pot} & \phi_{cam} & \theta_{cam} & \psi_{cam} \end{array}\right]}^{T}$$

The observation model that maps the state vector to the measurement vector is obtained as follows:

(67) $$h\left({\hat{x}}_{\left(k|k-1\right)}\right)={\left[\begin{array}{ccccccc} -\sin\theta & \cos\theta \sin\phi & \cos\phi \cos\theta & \theta & \phi & \theta & \psi \end{array}\right]}^{T}$$

The observation transition matrix given in (58) is obtained as follows:

(68) $$H_{k}=\left[\begin{array}{cccccc} 0 & -\cos\theta & 0 & 0 & 0 & 0\\ \cos\phi \cos\theta & -\sin\phi \sin\theta & 0 & 0 & 0 & 0\\ -\cos\theta \sin\phi & -\cos\phi \sin\theta & 0 & 0 & 0 & 0\\ 0 & 1 & 0 & 0 & 0 & 0\\ 1 & 0 & 0 & 0 & 0 & 0\\ 0 & 1 & 0 & 0 & 0 & 0\\ 0 & 0 & 1 & 0 & 0 & 0 \end{array}\right]$$

The measurement noise covariance matrix is obtained as follows:

(69) $$R_{k}=\left[\begin{array}{ccccccc} r_{{\mathrm{acc}}_{x}} & 0 & 0 & 0 & 0 & 0 & 0\\ 0 & r_{{\mathrm{acc}}_{y}} & 0 & 0 & 0 & 0 & 0\\ 0 & 0 & r_{{\mathrm{acc}}_{z}} & 0 & 0 & 0 & 0\\ 0 & 0 & 0 & r_{\theta_{pot}} & 0 & 0 & 0\\ 0 & 0 & 0 & 0 & r_{\phi_{cam}} & 0 & 0\\ 0 & 0 & 0 & 0 & 0 & r_{\theta_{cam}} & 0\\ 0 & 0 & 0 & 0 & 0 & 0 & r_{\psi_{cam}} \end{array}\right]$$

The updated state estimate given in (53) is obtained as follows:

(70) $${\hat{x}}_{\left(k|k\right)}={\left[\begin{array}{cccccc} \phi_{\mathrm{updated}} & \theta_{\mathrm{updated}} & \psi_{\mathrm{updated}} & {{\mathrm{bias}}_{x}}_{\mathrm{updated}} & {{\mathrm{bias}}_{y}}_{\mathrm{updated}} & {{\mathrm{bias}}_{z}}_{\mathrm{updated}} \end{array}\right]}^{T}$$

## 7. Results

First, we discuss the intermediate outcomes of identifying the aircraft skeleton from both side and top images, as the accuracy of attitude estimation heavily relies on the precise positioning of the corner points within the aircraft model skeleton.

**Figure 7:**
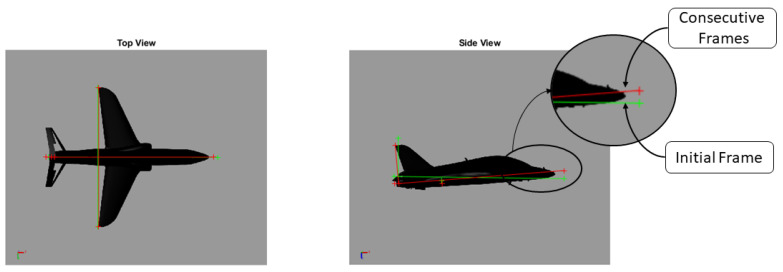
Initial frame and consecutive frame [16] (reprinted with permission from the American Institute of Aeronautics and Astronautics, Inc.).

The utilisation of Harris corner detection to identify corner points introduces varying errors in the pixel coordinates (u,v), leading to inaccuracies in the Euler angle estimation. The errors derived from Harris detection, exemplified by a single frame, are showcased in Table 1. Additionally, Figure 7 illustrates the corner points and aircraft model skeleton in the pixel coordinates, with green and red colours representing the initial frame and a consecutive frame, respectively. A notable limitation of applying corner detection algorithms to the current CAD model design and lighting setup is that the right-wing tip falls into a blind spot and remains invisible to the camera unless the aircraft model exhibits roll by more than 5 degrees. Additionally, the left wing tip is generally obscured and only becomes visible when the model exhibits extensive motions across all three degrees of freedom.

**Figure 8:**
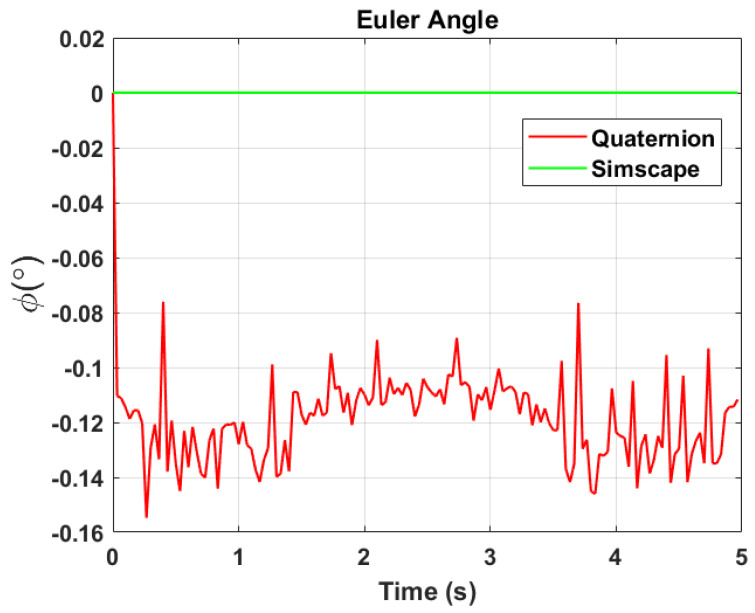
Euler angle, ϕ.

**Figure 9:**
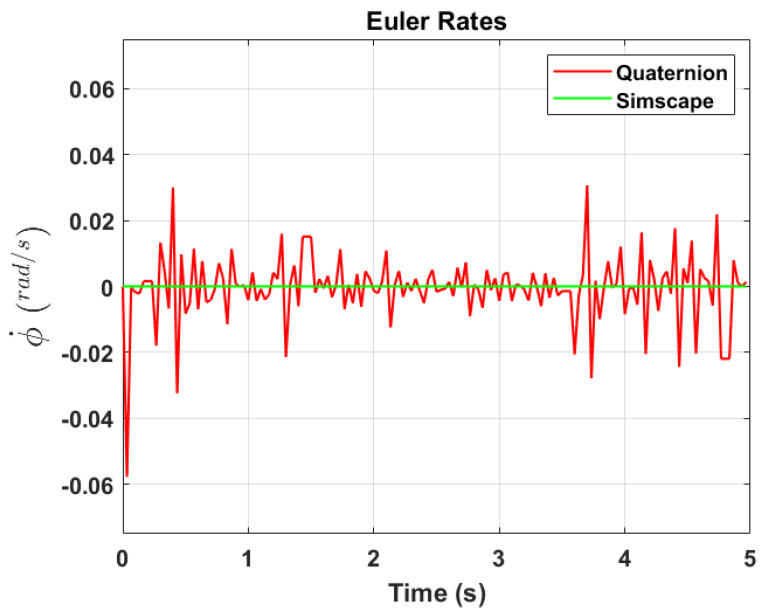
Euler rate, $\dot{\phi }$.

**Figure 10:**
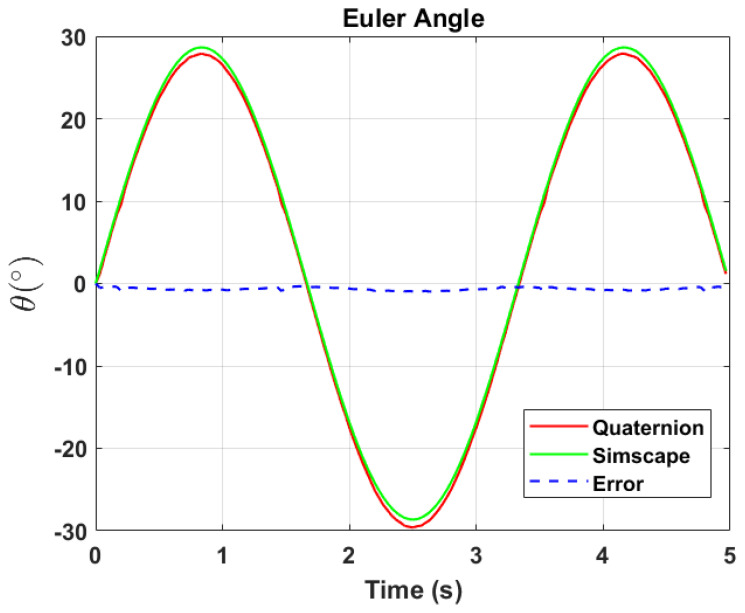
Euler angle, θ.

**Figure 11:**
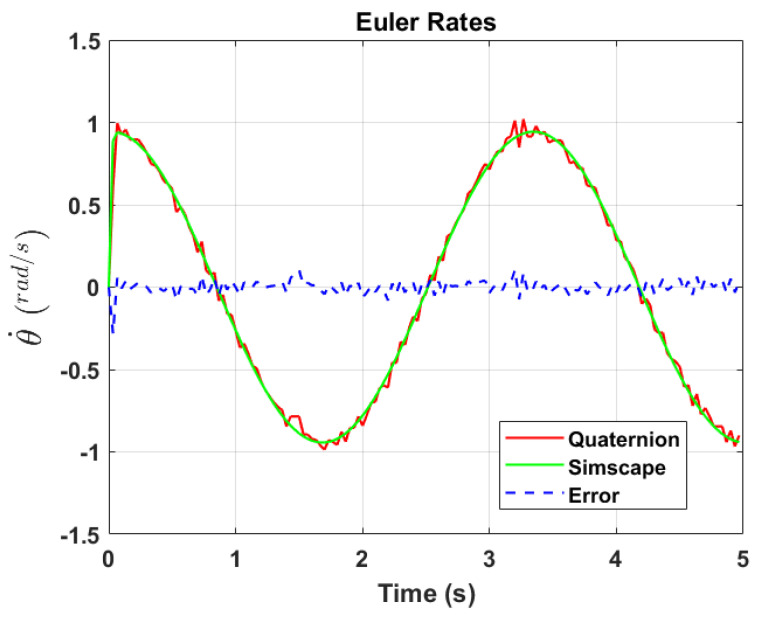
Euler rate, $\dot{\theta }$.

**Figure 12:**
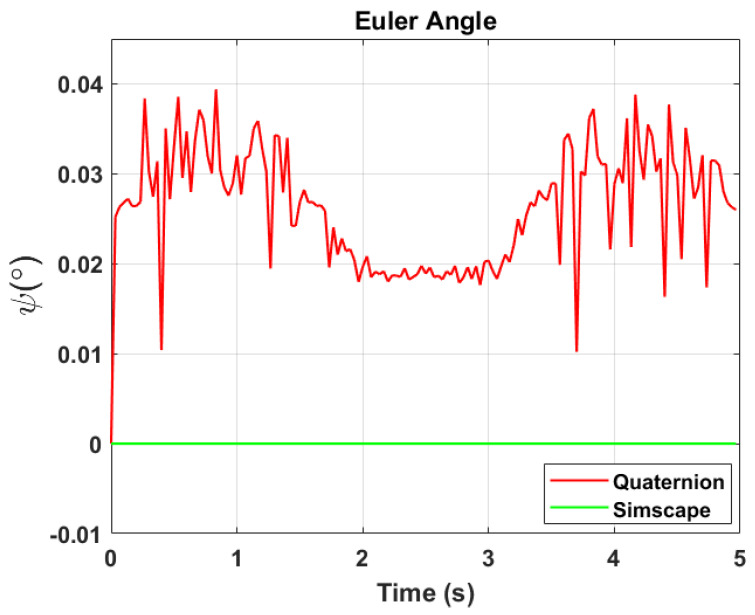
Euler angle, ψ.

**Figure 13:**
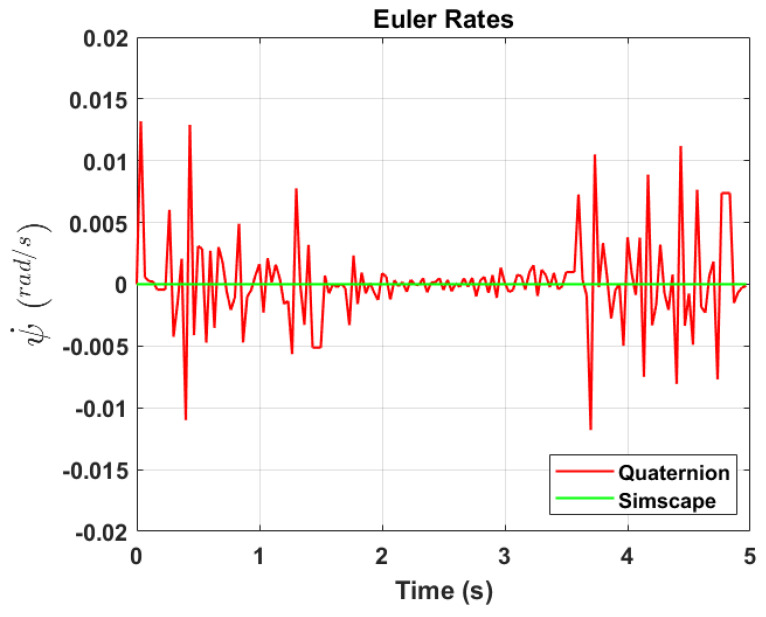
Euler rate, $\dot{\psi }$.

The transformation of 2D pixel coordinates to 3D coordinates of the aircraft model skeleton as described in Section 3.3 is illustrated in Figure 4. This figure demonstrates the accuracy of the customised transformation from 2D to 3D coordinates, specifically tailored for this orthogonal stereo setup, in successfully converting the aircraft skeleton points from the top and side camera views into a unified 3D world coordinate system. The rotation matrix obtained using (30) has a determinant of 1. This indicates that the matrix represents a pure rotation without any scaling effects. Figure 8, Figure 9, Figure 10, Figure 11, Figure 12 and Figure 13 illustrate the ϕ, θ, ψ, $\dot{\phi }$, $\dot{\theta }$, and $\dot{\psi }$ results from our proposed vision method and compare them to the Simscape data. Table 2 provides a comprehensive numerical representation of accuracy defined as the degree to which the estimated angles from our proposed method match the ground truth values represented by Simscape results, expressed as an error range. The following interpretations could be made from the results obtained from the quaternions:

1. The results of pitch angle (θ) indicate that there is no consistent bias towards underestimation or overestimation in these angles. However, the results of roll angle (ϕ) indicate a consistent bias towards underestimation and the results of yaw angle (ψ) indicate a consistent bias towards overestimation. Irrespective of the bias, the results follow a pattern of the input of the Simscape model, which is a sin wave.
2. The magnitude of the errors suggests that the quaternion method is accurate in estimating angles, with ϕ and ψ having relatively small errors and θ having relatively larger errors.
3. The bias of $\dot{\phi }$, $\dot{\theta }$, and $\dot{\psi }$ do not have a consistent bias towards underestimation or overestimation.
4. The magnitudes of the error range of $\dot{\theta }$ have large errors while the $\dot{\phi }$ and $\dot{\psi }$ have small errors identical to the Euler angles results as described in [16].

Even though quaternions offer accuracy and stability, small errors in their components can still lead to significant deviations in Euler angles, especially near zero rotation angles. This is due to the sensitivity of trigonometric functions involved in converting quaternions to Euler angles. The inaccuracies in identifying Euler angles propagate to Euler rates during backward differentiation. A significant error of −0.2806 radians per second in the $\dot{\theta }$ occurs at the first time step, which is at 0+Δt, with the subsequent minimum error changing to −0.0818 radians per second, suggesting that the primary error occurs initially.

By computing the root mean square error (RMSE) for each estimate of phi, theta, and psi individually, and then aggregating these RMSE values, the percentage improvement of the quaternion method when compared to the direction cosine matrix employed in [16], is determined by using the following equation,

(71) $$\mathrm{percentage}=\left(\frac{{RMSE}_{\mathrm{DCM}}-{RMSE}_{\mathrm{Quat}}}{{RMSE}_{\mathrm{DCM}}}\right)100\%$$

The results indicate a 45.41% improvement in the accuracy of the quaternion method for attitude estimation of the CAD model compared to using the direction cosine matrix method.

**Figure 14:**
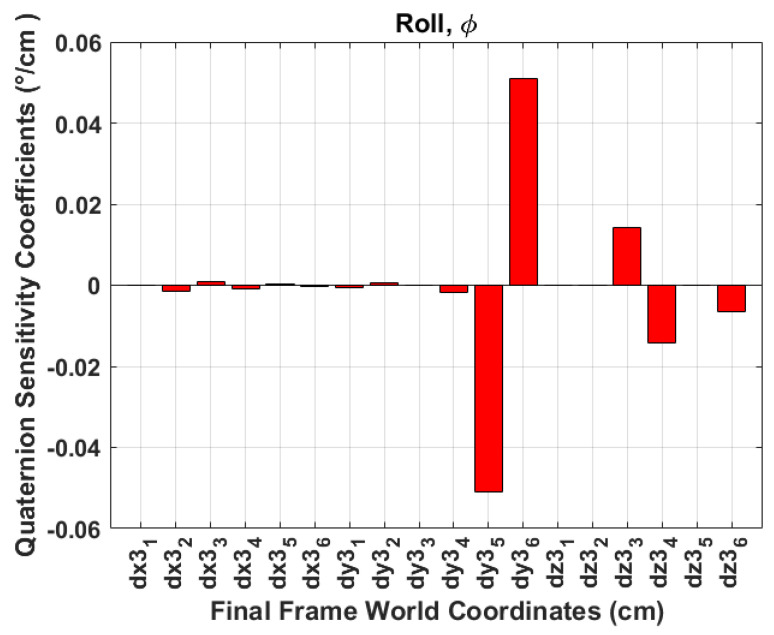
Sensitivity analysis roll.

**Figure 15:**
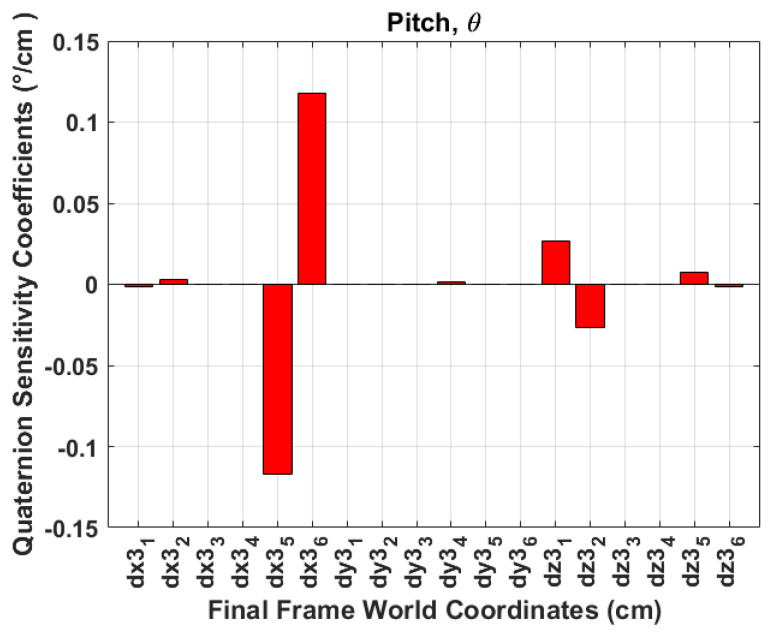
Sensitivity analysis theta.

**Figure 16:**
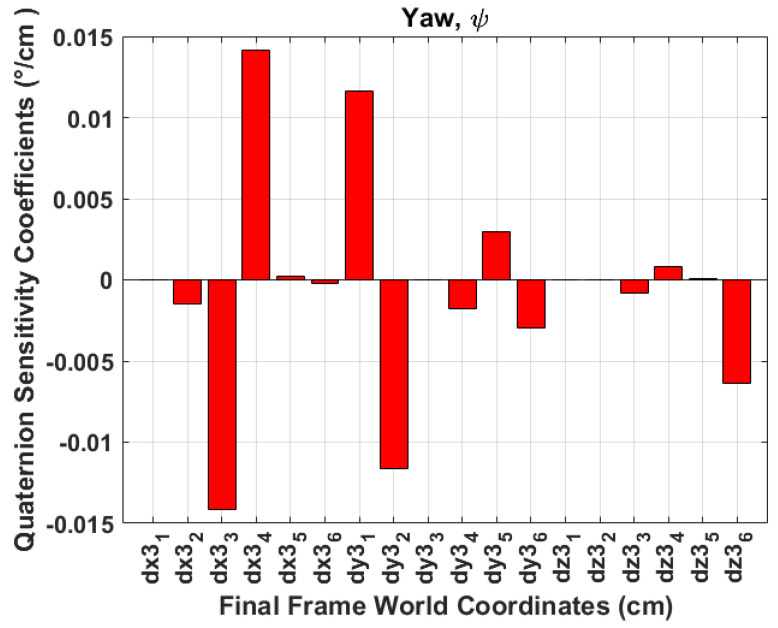
Sensitivity analysis yaw.

A sensitivity analysis is performed to evaluate the system’s robustness and its ability to maintain performance under varying errors in the identification of aircraft skeleton points. This analysis aims to understand how errors in accurately identifying the aircraft skeleton corner points, as visualised in Table 1, propagate to the Euler angle errors. Additionally, to further analyse the first-order changes in the Euler angles, random numbers between −1 and 1 are chosen as $\Delta P_{3}$. Table 3 depicts the obtained first-order change of functions. The results indicate the following:

1. The world coordinate points (${Y_{3}}_{\left(2,5\right)}$, ${Y_{3}}_{\left(2,6\right)}$, ${Z_{3}}_{\left(3,3\right)}$, and ${Z_{3}}_{\left(3,4\right)}$) have a high impact on the change in ϕ angles. These points are the *Y*-coordinates of the tail length and *Z*-coordinates of wing tips. This is visualised in Figure 14, where **dx3**_1_, ⋯, **dy3**_1_, ⋯, **dz3**_1_ represent $\partial {X_{3}}_{\left(1,1\right)}$, ⋯, $\partial {Y_{3}}_{\left(1,1\right)}$, ⋯, $\partial {Z_{3}}_{\left(1,1\right)}$, which are the aircraft skeleton points in consecutive frames as defined in (23).
2. Figure 15 indicates that the world coordinate points (${X_{3}}_{\left(1,5\right)}$, ${X_{3}}_{\left(1,6\right)}$) have a significant impact on the change of θ angles. These points are the *X*-coordinates of the tail length and the points ${Z_{3}}_{\left(3,1\right)}$, and ${Z_{3}}_{\left(3,2\right)}$ have slightly minimal impact on the change in θ angles. These points are the *Z*-coordinates of the fuselage length.
3. Figure 16 depicts that the points (${X_{3}}_{\left(1,3\right)}$, ${X_{3}}_{\left(1,4\right)}$, ${Y_{3}}_{\left(2,1\right)}$, and ${Y_{3}}_{\left(2,2\right)}$) have a high impact on the ψ angles. These are the *X*-coordinates of the wing tips and the *Y*-coordinates of the fuselage length.
4. Any inaccuracies or disturbances in the identification of $P_{3}$ points tend to amplify the roll and yaw angle and affect the accuracy or stability in ϕ and ψ estimation.
5. The results obtained from quaternion measurements were corroborated by the sensitivity analysis, affirming their reliability in obtaining ϕ,θ,ψ angles. Additionally, the sensitivity analysis reveals that the pitch angle exhibits the highest susceptibility to noise or inaccuracies in aircraft skeleton points, followed by the roll angle, while the yaw angle demonstrates relatively lower sensitivity.

**Figure 17:**
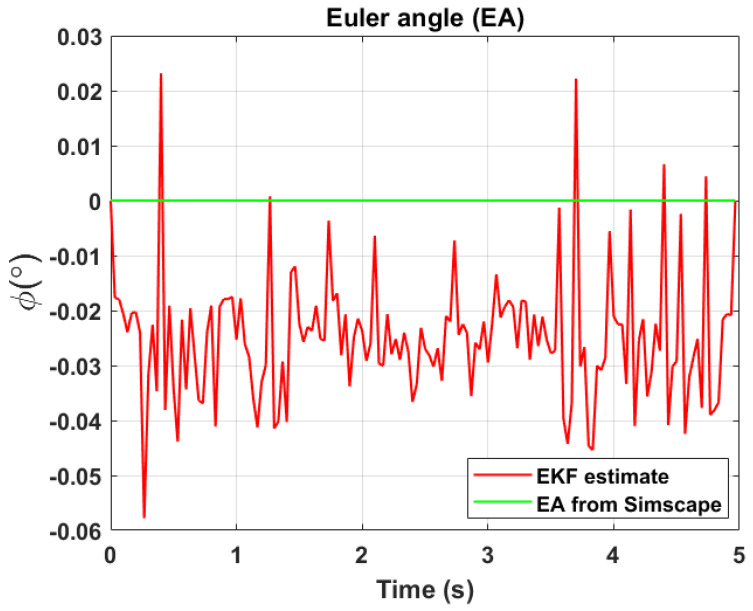
EKF Euler angle, ϕ.

**Figure 18:**
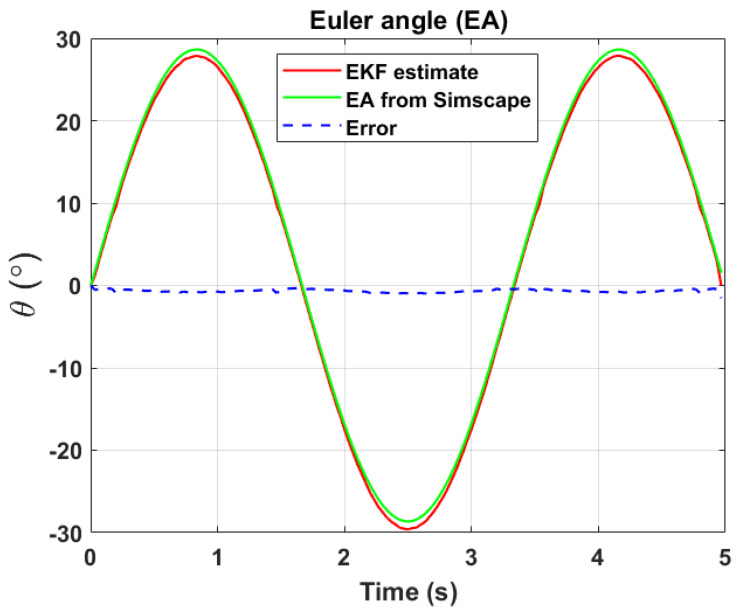
EKF Euler angle, θ.

**Figure 19:**
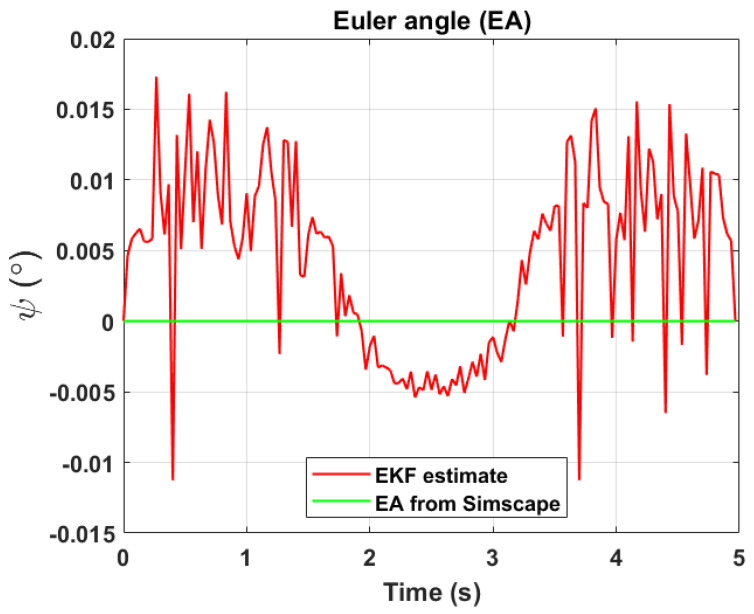
EKF Euler angle, ψ.

**Figure 20:**
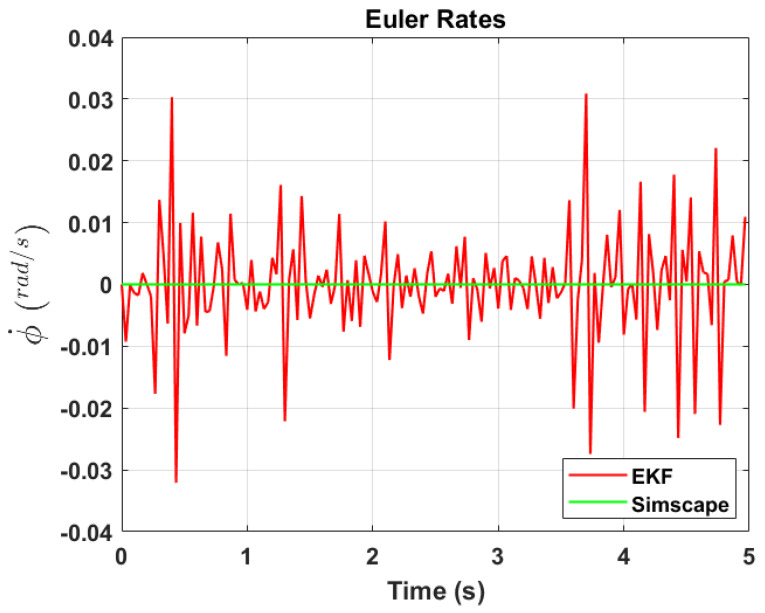
EKF Euler rate, $\dot{\phi }$.

**Figure 21:**
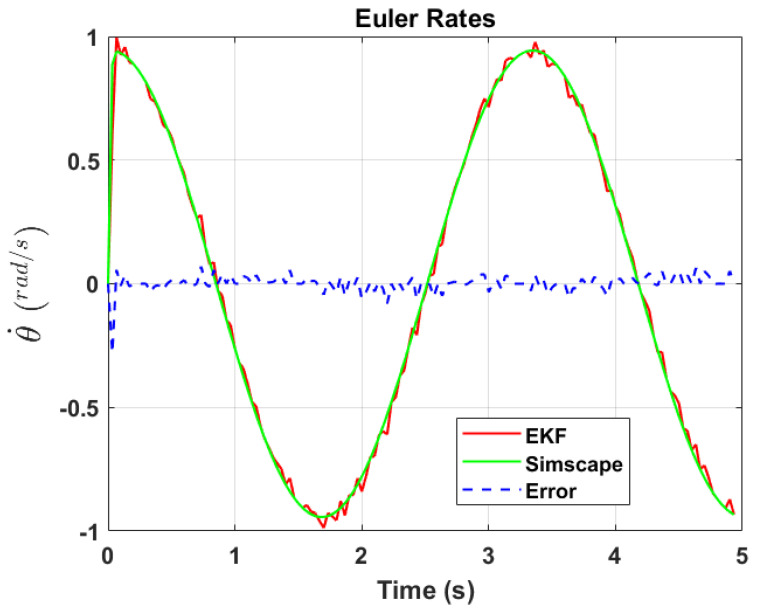
EKF Euler rate, $\dot{\theta }$.

**Figure 22:**
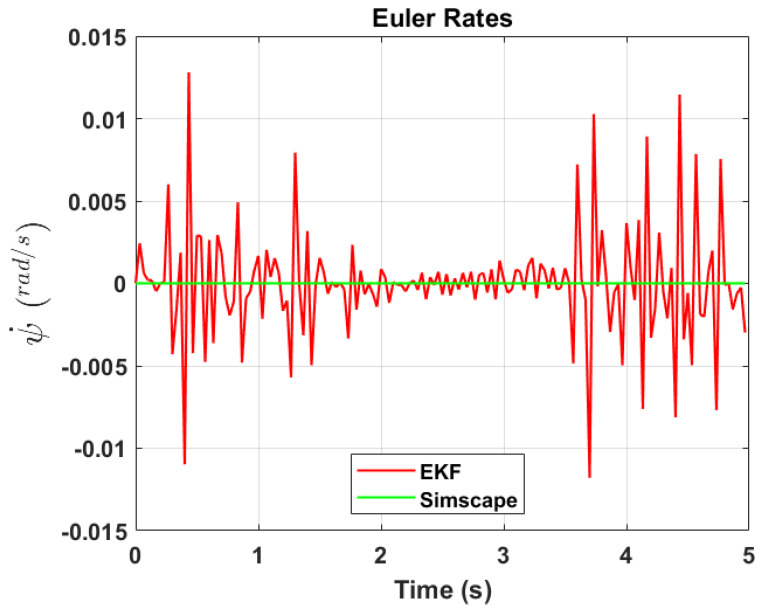
EKF Euler rate, $\dot{\psi }$.

Figure 17, Figure 18 and Figure 19 depict the results of EKF state (ϕ,θ,ψ) estimation, respectively. The dynamic measurements $\left(\dot{\phi },\dot{\theta },\dot{\psi }\right)$ obtained through backward differentiation of EKF’s state estimates are illustrated in Figure 20, Figure 21 and Figure 22, respectively. Table 4 provides a comprehensive numerical representation of the error ranges. Comparing the EKF results to the quaternion results in Table 2 indicates that the EKF estimation of Euler angles is consistent with the expected error range from quaternions. Comparing the EKF results with the Simscape results indicates the following:

1. A comprehensive and reliable estimate of the system state is derived by the EKF fusion process that effectively integrates information from different sensors. This indicates a reliable fusion by the EKF.
2. The EKF is robust to variations, noise, and uncertainties present in the gyroscope, accelerometer, and potentiometer data. This demonstrates the ability of EKF to effectively handle sensor measurements and provide state estimation even in challenging conditions.
3. The results from EKF perform as validation for the sensor fusion algorithm implemented in the EKF. It indicates that the fusion process is functioning correctly.

## 8. Conclusions

A computer vision technique is proposed for the attitude estimation of a scaled aircraft model in dynamic wind tunnel testing without the placement of any markers on the model. This implementation is novel and has not been previously executed. The method implements a sequential process of Harris corner detection, Kanade–Lucas–Tomasi tracking, and quaternions to identify the Euler angles. The Harris detection method introduces inaccuracies in identifying the corner points of the aircraft model skeleton, thereby affecting the estimation of Euler angles. A sensitivity analysis of the proposed approach was conducted using a first-order Taylor series. The implementation of quaternions over the direction cosine matrix for estimating Euler angles has shown a 45.41% improvement in accuracy. The quaternion method achieves an RMSE of 0.0101 rad/s, 0.0361 rad/s, and 0.0036 rad/s for the dynamic measurements of $\dot{\phi },\dot{\theta },$ and $\dot{\psi }$, respectively. Additionally, this method exhibits an accuracy of 98.08% for pitch rate ($\dot{\theta }$). Furthermore, by incorporating data from on-board sensors such as an IMU and a potentiometer gimbal, and integrating it with vision data using the extended Kalman filter, this method has achieved an RMSE of 0.0090 rad/s, 0.0262 rad/s, and 0.0034 rad/s for the dynamic measurements of $\dot{\phi },\dot{\theta },$ and $\dot{\psi }$, respectively. This method exhibits an improved accuracy of 98.61% for the estimation of pitch rate ($\dot{\theta }$).

Additional verification of the method through application to lateral and longitudinal angle changes will be performed, along with full integration with other measurement techniques. While the approach developed here works satisfactorily without markers, making this a challenging exercise, the inclusion of markers should improve accuracy and reliability. The system will also be integrated into a longitudinal 3-degree-of-freedom rig that is currently being developed [41,42].

## Figures and Tables

**Table 1:** Harris detection method errors.

Corner Points	X Pixel Error	Y Pixel Error
Side View Tail P1	0.880	1.312
Top View Wing P1	2.052	0.052
Top View Wing P2	1.060	0.583
Top View HS	2.052	±1.052
Side View HS P1	1.229	−1.171
Side View HS P2	1.257	−1.047

**Table 2:** Error range of Euler angles and rates from quaternions.

Angles	Error Range (°)	Rates	Rates Error (rad/s)
ϕ	$\left[-0.1548,\;0\right]$	$\dot{\phi }$	$\left[-0.0578,\;0.0307\right]$
θ	$\left[-0.9924,\;0\right]$	$\dot{\theta }$	$\left[-0.2806,\;0.1041\right]$
ψ	$\left[0,\;0.0394\right]$	$\dot{\psi }$	$\left[-0.0118,\;0.0134\right]$

**Table 3:** First-order change of the functions.

Euler Angles	Change in Angles (°)
ϕ	0.0194
θ	−0.0569
ψ	0.0111

**Table 4:** Error range of EKF attitude estimation.

Angles	Error Range (°)	Rates	Rates Error (rad/s)
ϕ	$\left[-0.0578,\;0.0232\right]$	$\dot{\phi }$	$\left[-0.0321,\;0.0309\right]$
θ	$\left[-0.9928,\;0\right]$	$\dot{\theta }$	$\left[-0.2806,\;0.0701\right]$
ψ	$\left[-0.0113,\;0.0173\right]$	$\dot{\psi }$	$\left[-0.0118,\;0.0128\right]$

## Data Availability

The MATLAB software utilised for processing both on-board and off-board data, as well as for attitude estimation, is available in the Cranfield Online Research Data repository (https://doi.org/10.17862/cranfield.rd.25700061).

## Abbreviations

The following abbreviations are used in this manuscript:

DWT	dynamic wind tunnel
DOF	degree-of-freedom
MEMS	micro-electro-mechanical system
RLG	ring laser gyro
UAV	unmanned aerial vehicle
IMU	inertial measurement unit
DCM	direction cosine matrix
CAD	computer-aided design
DLT	direct linear transformation
PnP	perspective-n-point
ROI	region of interest
EKF	extended Kalman filter
